# A multi-omics analysis of viral nucleic acid poly(I:C) responses to mammalian testicular stimulation

**DOI:** 10.1007/s44154-023-00146-6

**Published:** 2024-02-01

**Authors:** Donghui Yang, Wenping Wu, Qizhong Lu, Yaling Mou, Wenbo Chen, Shicheng Wan, Mengfei Zhang, Congliang Wang, Xiaomin Du, Na Li, Jinlian Hua

**Affiliations:** 1 Present Address: College of Veterinary Medicine, Shaanxi Centre of Stem Cells Engineering & Technology, Northwest A&F University, No. 3rd, Taicheng Road, Yangling, 712100 Shaanxi China; 2 State Key Laboratory of Biotherapy and Cancer Center, West China Hospital, Sichuan University, Chengdu, 610041 China; 3 Shaanxi Provincial Engineering and Technology Research Center of Cashmere Goats, College of Life Sciences, Yulin University, Yulin, 719000 Shaanxi China

**Keywords:** Orchitis, Poly(I:C), Proteomic, Metabolomic, Homeostasis

## Abstract

The male reproductive system has a standard immune response regulatory mechanism, However, a variety of external stimuli, including viruses, bacteria, heat, and medications can damage the testicles and cause orchitis and epididymitis. It has been shown that various RNA viruses are more likely to infect the testis than DNA viruses, inducing orchitis and impairing testicular function. It was found that local injection of the viral RNA analog poly(I:C) into the testes markedly disrupted the structure of the seminiferous tubules, accompanied by apoptosis and inflammation. Poly(I:C) mainly inhibited the expression of testosterone synthesis-associated proteins, STAR and MGARP, and affected the synthesis and metabolism of amino acids and lipids in the testis. This led to the disruption of the metabolite levels in the testis of mice, thus affecting the normal spermatogenesis process. The present study analyzed the acute inflammatory response of the testis to viral infection using a multi-omics approach. It provides insights into how RNA virus infection impairs testicular function and offers a theoretical basis for future studies on immune homeostasis and responses under stress conditions in male reproduction.

**Supplementary Information:**

The online version contains supplementary material available at 10.1007/s44154-023-00146-6.

## Introduction

Mammalian testicles have two main functions: the production of sperm and the synthesis of male hormones. Spermatogenesis, located in the seminiferous tubules, is accomplished by the Spermatogenic stem cells (SSCs) through self-renewal and differentiation; SSCs play a vital role in the maintenance of high spermatogenesis (Ibtisham and Honaramooz 2020). The Sertoli cells that extending from the basement membrane to the lumen of the seminiferous tubules, form the spermatogenic epithelium and make up the main structures. These cells also create the blood-testicular barrier (BTB), which is a barrier between testicular germ cells and the immune system (Mao et al. 2020). The BTB limits the contact between germ cell antigens and immune cells, preventing the entry of antibodies into the seminiferous tubules; this results in the loss of immune surveillance function (Wu et al. 2019). In mammals, the blood-brain barrier (BBB) (Terstappen et al. 2021) and blood-fetal barrier (BPB) (Stein-Streilein and Caspi 2014) are similar to the function of the BTB. The Sertoli cells not only provide nutrients to germ cells but also offer immune protection (Chen et al. 2016). Androgens, on the other hand, are synthesized by various cells in the testicular interstitium (Garza et al. 2022), including Leydig cells, macrophages, and lymphocytes.

Viruses, such as Zika virus (ZIKV) (Shang et al. 2022), Human immunodeficiency virus (HIV) (Wu et al. 2021), Ebola virus (Xia 2020), and mumps virus (MuV) (Wu et al. 2017), act as potent stimulants that are capable of infecting not only the testicles but also the entire male reproductive tract through the bloodborne route. This is because viruses can induce cells to produce a large amount of interferon and express a variety of antiviral proteins, which play a significant role in the testicles’ resistance to viral infections (Le Tortorec et al. 2020). BTB, which protects germ cells located in the seminiferous tubules from immune cells in the interstitium, and the immune protective functions of Sertoli cells, which may contribute to immune privilege, are additional mechanisms that safeguard the testes (Fijak et al. 2018). Moreover, Leydig cells, being the most abundant cell in the testicular interstitium, secrete numerous cytokines that have a crucial role in the natural immune response of the testes and the maintenance of the immune immunity state, primarily by regulating the proliferation and differentiation of various cells (Shen et al. 2021). However, despite these insights, the precise connection between viral infection and male infertility remains incompletely understood (Hu et al. 2015).

In this study, a multi-omics method was used to analyze the acute inflammatory response of the testis after viral infection, explicitly using poly(I:C), a commonly used synthetic analog of double-stranded (ds) RNA viruses for mimicking viral infections (Han et al. 2023; Helou et al. 2023). The study’s objective was to explore a new mechanism of immune homeostasis triggered by the mammalian reproductive system after viral infection. The findings of this study provide a theoretical basis for future research on male reproductive immune homeostasis.

## Results

### Poly(I:C)-induced testicular damage in mice

**Fig. 1:**
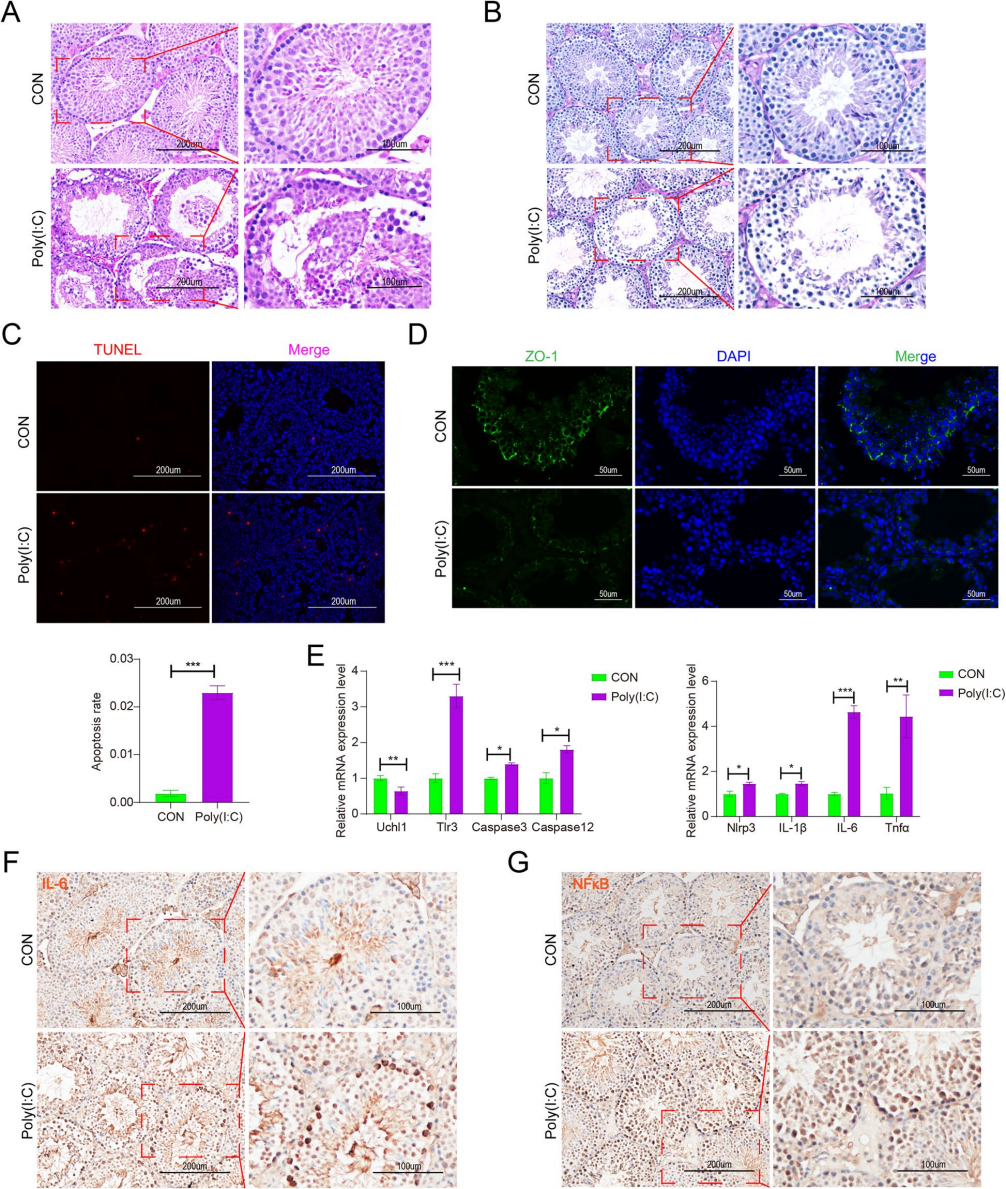
Effect of Poly(I:C) on mouse testes. **A**, **B** Testicular tissue was stained using HE staining and PAS staining. Six 8-week-old ICR mice (*n* = 5) received local injections of Poly(I:C) in six testes, while an equal volume of PBS was injected into the contralateral testes as a control. **C** Testicular tissues were stained using TUNEL staining. **D** IF staining of ZO-1 in testis. **E** mRNA levels of *Uchl1*, *Tlr3*, *Caspase3*, *Caspase12*, *Nlrp3*, *IL-1β*, *IL-6*, and *Tnfα* genes were measured using RT-qPCR. **F**, **G** Immunohistochemical staining of testicular tissue for IL-6 and NFκB. (Data are presented as means±SD and represent three independent repetitions. *: *P* < 0.05; **: *P* < 0.01; ***: *P* < 0.001)

In the present study, poly(I:C), a dsRNA viral mimic, was used to induce a model of testicular inflammation to investigate its effect on the immune microenvironment of the testis. Injection of poly(I:C) resulted in the disruption of testicular structure, detachment of germ cells in the convoluted seminiferous tubules from the basement membrane site, and hemolytic infiltration of the testicular interstitium, as revealed by HE staining (Fig. 1A). PAS staining also further showed loose arrangement of germ cells in the varicocele of Poly(I:C) group and accumulation of glycogen in the testicular interstitium (Fig. 1B). TUNEL staining showed that apoptosis in the poly(I:C) group was significantly upregulated compared with the control (Fig. 1C). BTB serves as a special immune barrier in the testis to protect germ cells from immune responses, we examined the expression of ZO-1, a structural protein of the BTB, and the immunofluorescence results showed that the expression of ZO-1 was downregulated in poly(I:C) (Fig. 1D), which indicated that the structure of the BTB was disrupted. We further examined the expression levels of apoptosis and inflammatory factors, focusing on the toll-like receptor 3 (Tlr3) pathway activation caused by poly(I:C). Ubiquitin C-Terminal Hydrolase L1 (Uchl1) expression was mainly observed in mouse testis SSCs and partially in Sertoli cells. After poly(I:C) infection, there was a downregulation of Uchl1 expression, while the expression of Tlr3, Caspase3, Caspase12, NOD-like receptor thermal protein domain associated protein 3 (Nlrp3), Interleukin-1β (IL-1β), IL-6, and Tumor necrosis factor alpha (Tnfα) was significantly upregulated. These findings indicate that poly(I:C) activates the Tlr3 signaling pathway, leading to apoptosis and inflammation (Fig. 1E). At the histologic level, we examined the expression of inflammatory factors IL-6 (Fig. 1F) and NFκB (Fig. 1G) in the testis, which were significantly higher compared with the control group. Overall, the results suggest that the poly(I:C)-induced testicular inflammation model was successfully established and triggered testicular cell apoptosis accompanied by an onset of the inflammatory response.

### Proteomic analysis of poly(I:C)-induced orchitis in mice

**Fig. 2:**
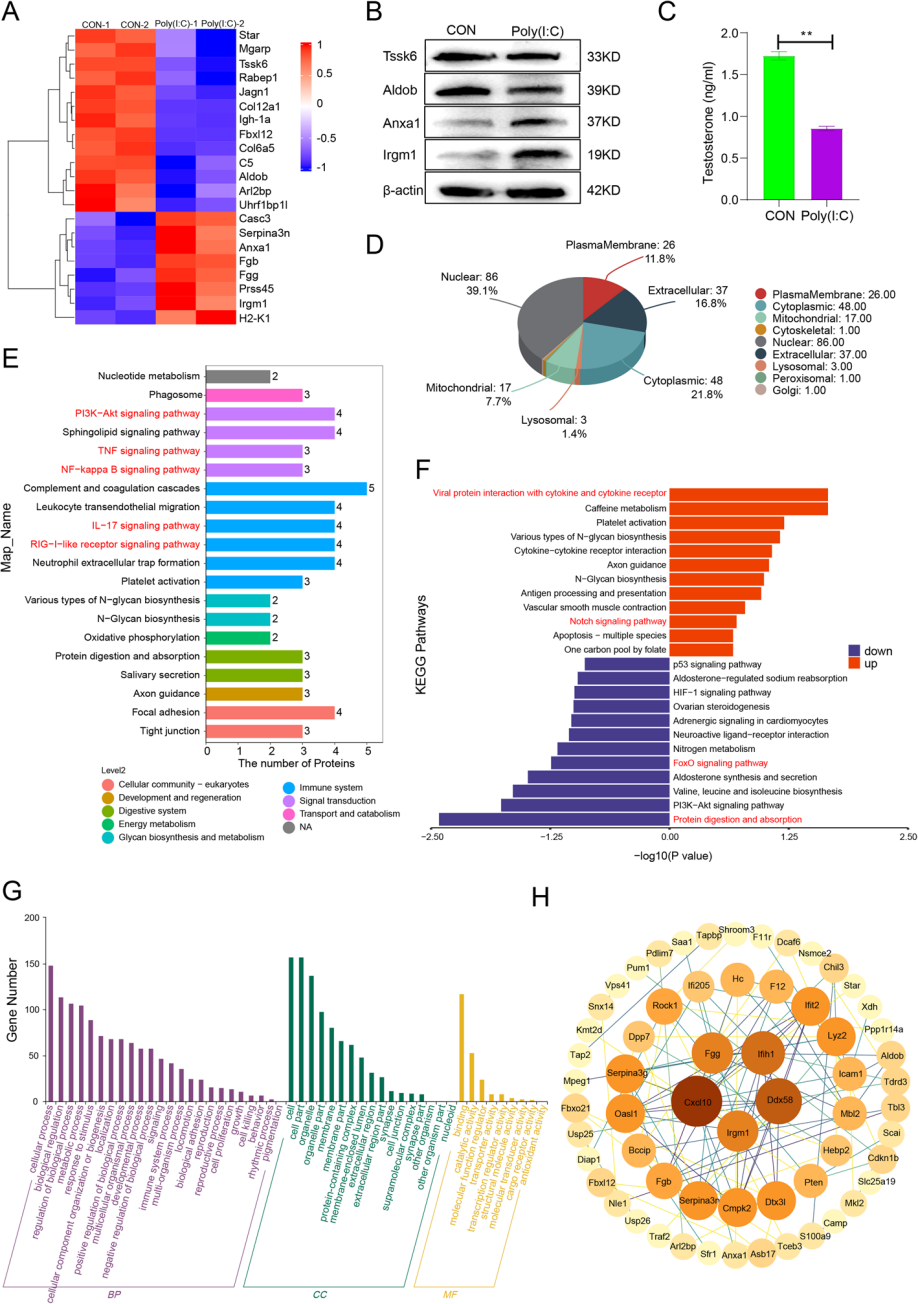
Proteomic analysis of Poly(I:C)-induced testicular inflammation. **A** Heatmap of significantly differentially expressed proteins (Fold change > 2 and *P* value< 0.05); (**B**) Western blot detected the changes of key proteins (Tssk6, Aldob, Anxa1 and Irgm1) in testicular tissue. **C** Serum testosterone levels in mice. **D** Subcellular localization analysis of all differentially expressed proteins; (**E**) Annotation and attribution of differentially proteins pathway enriched; (**F**) Analysis of up- and down-regulation of KEGG pathway enriched by differentially proteins; (**G**) Functional annotation of GO for all differentially expressed proteins; (**H**) A protein-protein interaction (PPI) network was constructed for the differentially expressed proteins

To investigate the specific mechanism of poly(I:C)-induced testicular inflammation, proteomic sequencing was performed on the poly(I:C) model and control groups. Screening criteria of fold change > 2 and *P* value< 0.05 were used to obtain the significantly differentially expressed proteins (Fig. 2A); the result indicated that poly(I:C) suppresses the expression of testosterone-related proteins while upregulating the expression of immune-related factors. Western blot experiments detected the expression levels of key proteins, in which the expression of TSSK6, Aldob was down-regulated and the expression of Anxa1, Irgm1 was up-regulated, which was consistent with the sequencing results (Fig. 2A, B). Meanwhile, we examined the levels of testosterone hormone in the serum of mice. The results showed that the testosterone levels in the Poly(I:C) group were significantly down-regulated compared to the control (Fig. 2C). The subcellular localization of the differentially expressed proteins was found to be mainly distributed in the nuclear (39.1%), cytoplasmic (21.8%), extracellular (16.8%), plasma membrane (11.8%), mitochondrial (7.7%), and lysosomal (1.4%) (Fig. 2D). Further analysis of the differential protein pathways and their corresponding biological functions revealed a focus on the immune system and signal transduction branches (Fig. 2E). Activation of several inflammatory signaling pathways, such as TNFα, NFκB, and Interleukin-17 (IL17), was observed. To better understand the significance of the differential pathways associated with the proteins, a KEGG pathway enrichment analysis was performed. The results showed significant upregulation of inflammation-related pathways, such as viral protein interaction with cytokine and its receptor, and downregulation of protein digestion and absorption, and aldosterone synthesis and secretion (Fig. 2F). GO function annotation categorized the proteins into Biological Processes (BP), Molecular Function (MF), and Cellular Component (CC). Enrichment analysis showed significant involvement in cellular and metabolic processes, as well as binding and catalytic activity at the Molecular Function level. In the CC category, the proteins were highly enriched in cellular, organelle, and membrane components (Fig. 2G). Analysis of the protein-protein interaction network (PPI) identified Fgg and Irgm1 proteins as core targets in the network (Fig. 2H). These results suggest that poly(I:C) activates inflammation-related pathways in the testes and inhibits the synthesis of testosterone-related proteins.

### Metabolomic analysis of poly(I:C)-induced orchitis in mice

**Fig. 3:**
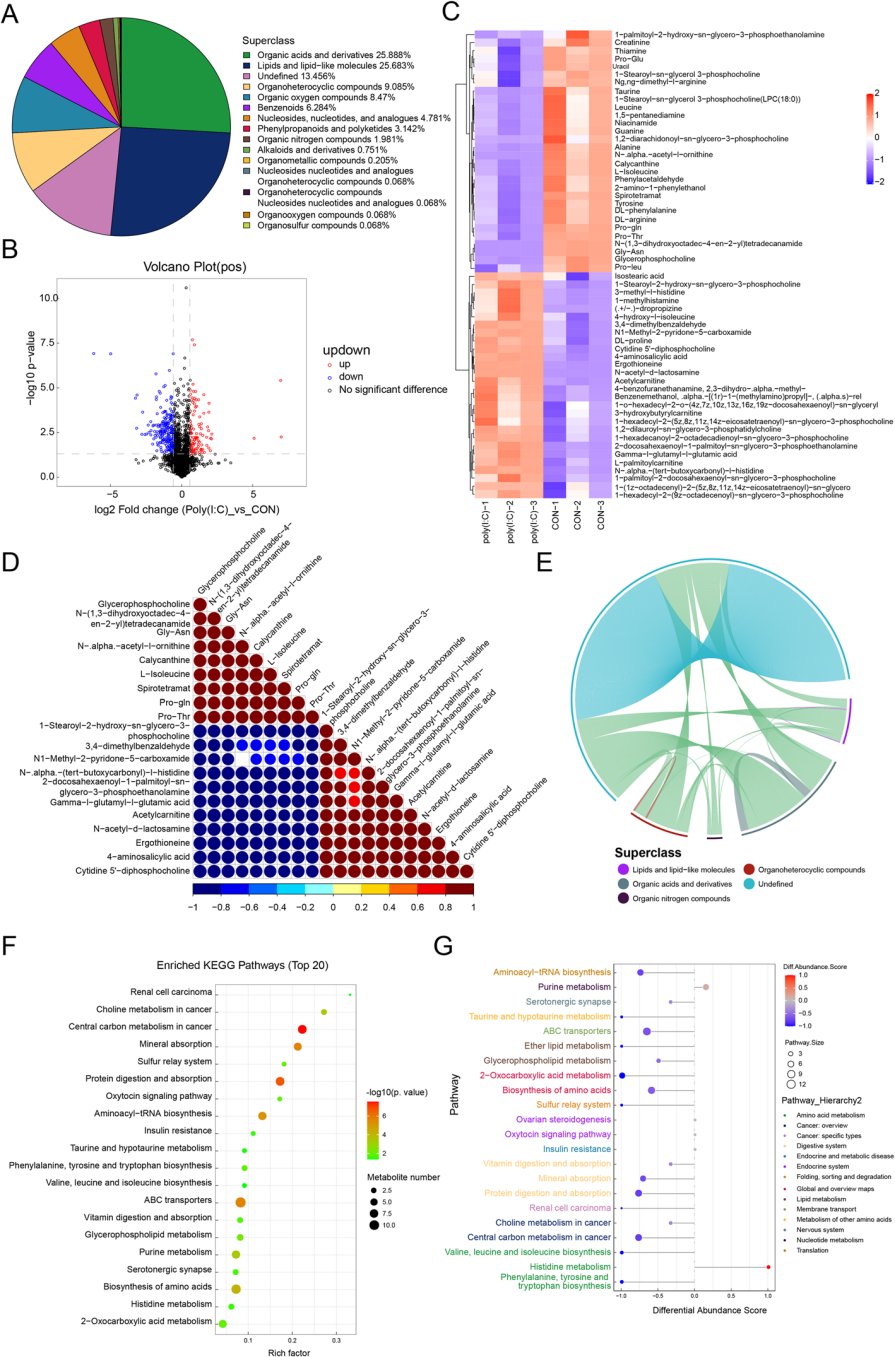
Metabolomic analysis of Poly(I:C)-induced testicular inflammation. **A** Chemical classification analysis showed the number of identified metabolites for each category as a percentage; (**B**) A volcano plot was generated for significantly different metabolites in positive ion mode; (**C**) A heatmap was created to cluster significant difference metabolites in positive ion mode; (**D**) The correlation between significant difference metabolites in positive ion mode was visualized using a correlation heatmap; (**E**) Positive ion mode chordal plot; (**F**) Bubble plot showed the top 20 most significant KEGG pathway maps; (**G**) Differential abundance scores (DA score) plots for all enriched metabolic pathways

In this part, we performed a metabolomic analysis of poly(I:C)-induced orchitis, considering metabolomics is the closest to the phenotype. A total of 1464 metabolites were identified by combining positive (Pos) and negative (Neg) ion patterns, with 930 being Pos and 534 being Neg. According to their chemical taxonomy attribution information, the identified metabolites were categorized. The largest proportions were organic acids and derivatives (25.888%) and Lipids and lipid-like molecules (25.683%) (Fig. 3A). Variations in the differential metabolites were depicted in volcano plots using Pos and Neg ion patterns (Fig. 3B, S1A), and the specific differential metabolites were displayed in heatmaps (Fig. 3C, S1B). The correlation matrix of the different metabolites revealed synergistic or mutually exclusive relationships (Fig. 3D, S1C), further illustrated by chord diagrams. The differential metabolites in the positive and negative ion modes were classified into five and six categories, respectively, with organic acids and derivatives being the most significant category in the pos ion mode, followed by Organic heterocyclic compounds (Fig. 3E, S1D). KEGG-enriched pathway analysis was performed on all the identified differential metabolites. The main enrichment pathways were protein digestion and absorption, ABC transporters, and biosynthesis of amino acids, which were consistent with proteome enrichment pathways (Fig. 3F). The Differential Abundance Score (DAS) analysis indicated that the pathways attributed to global and overview maps and digestive system were the most numerous and tended to be down-regulated overall. Histidine metabolism and Purine metabolism were the only upregulated pathways (Fig. 3G). Additionally, the network correlation between metabolites in the negative ion mode to depict the correlation between different metabolites (Fig. S1E), the network showed that uric acid and myristic acid are in the core metabolic network.

### Proteomic and metabolomic association analysis

**Fig. 4:**
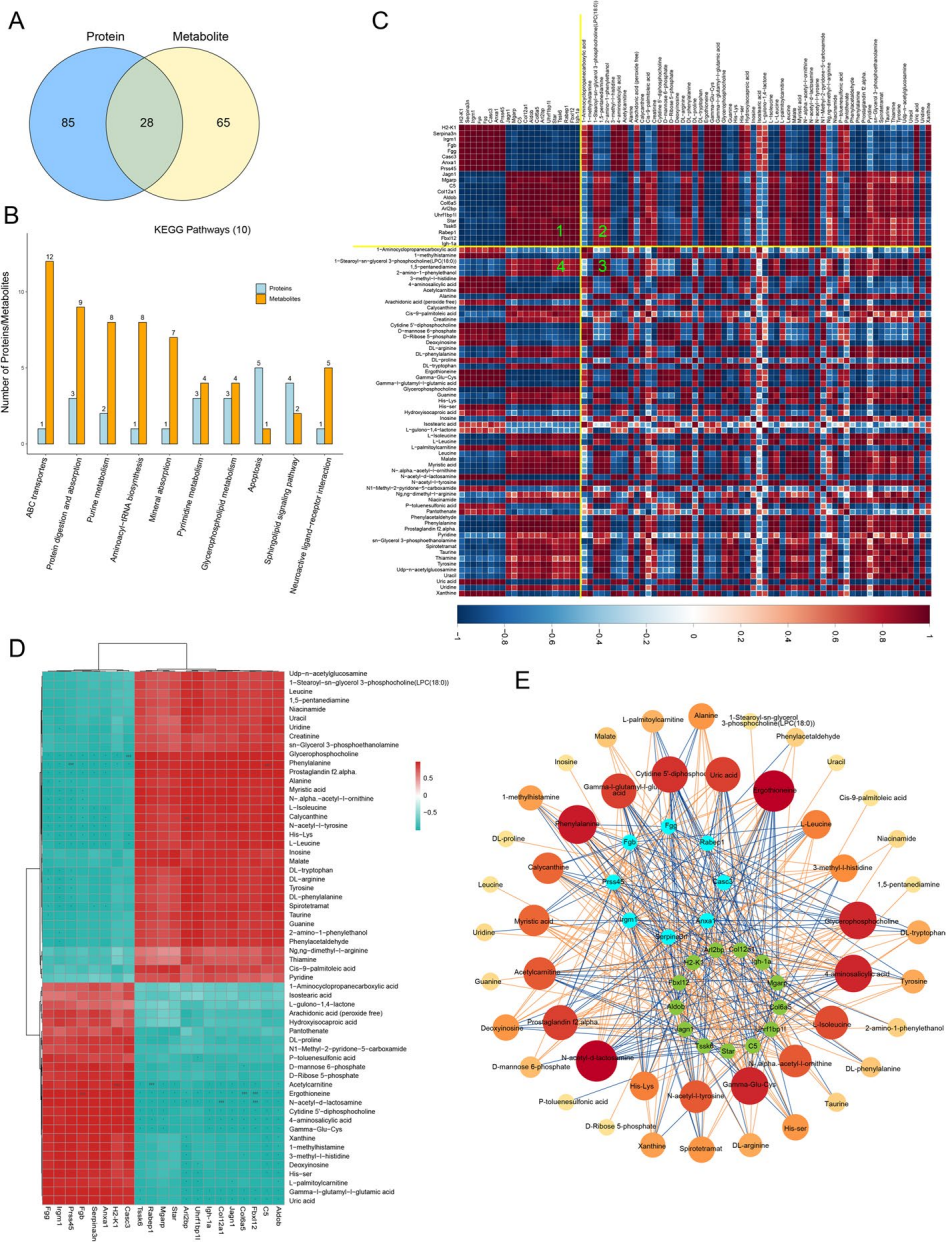
Combined proteomic and metabolomic analysis. **A** Venn plots were used to display the differential proteins and metabolites involved in pathways; (**B**) The top 10 pathways containing the highest number of proteins and metabolites together were identified. **C** A heat map was generated to show the correlation coefficients of significant differential proteins with significant differential metabolites. Positive correlation was indicated by red (r > 0), while negative correlation was indicated by blue (r < 0). The intensity of the color reflected the strength of the correlation; (**D**) A hierarchical clustering heatmap was created using Pearson correlation analysis to show the correlation between differential proteins and differential metabolites. Positive correlation was indicated by red (r > 0), while negative correlation was indicated by blue (r < 0). The intensity of the color reflected the strength of the correlation. *: *P* < 0.05; **: *P* < 0.01; ***: *P* < 0.001. **E** The network diagram displayed the correlation analysis between proteins and significantly different metabolites

In order to comprehensively compare the mechanism of change induced by poly(I:C) in the testis, we compared the metabolic pathways involved in differentially expressed proteins in the proteome with the metabolites differentially expressed in the metabolome. By analyzing Venn diagrams, it was found that there are 28 common metabolic pathways between the two omics. Additionally, there are 85 specific pathways identified in the proteome and 65 specific pathways identified in the metabolome (Fig. 4A). To further understand the relationship between identified proteins and metabolites, the top 10 pathways of KEGG with the highest number of identified proteins and metabolites were analyzed. Metabolic pathways such as Pyrimidine metabolism and Glycerophospholipid metabolism showed higher enrichment of differential proteins and metabolites (Fig. 4B). A correlation coefficients matrix heatmap was generated using the Pearson correlation analysis method. The heatmap was divided into four quadrants, showing the correlation between differential proteins and differential metabolites in quadrants 1 and 3, and the correlation between significant differential proteins and metabolites in quadrants 2 and 4 (Fig. 4B). To compare the expression patterns of significantly different proteins and metabolites, a Pearson correlation hierarchical clustering analysis was performed. Clusters of significantly different metabolites or proteins with similar expression patterns appeared in the same cluster (Fig. 4C). Finally, a network diagram illustrating the correlation analysis between significantly different proteins and metabolites was generated. The thickness of the lines represents the strength of the correlation, while red and blue colors represent positive and negative correlations, respectively (Fig. 4D).

## Discussion

One of the leading causes of male infertility is the destruction of the immune immunity mechanism of the testicles, which can be caused by viral or bacterial infection, stress, or genetic factors (Gan et al. 2023; Tsetsarkin et al. 2018; Yu et al. 2022). Orchitis and epididymitis are key factors contributing to male infertility, accounting for up to 15% of male fertility disorders (Fijak et al. 2018; Punab et al. 2017). When orchitis occurs, it disrupts the immune immunity status of the testicles, leading to a disordered homeostasis of the testicular microenvironment. This disruption is caused by the secretion of inflammatory factors, such as IL-6, IL-1β, TNFα, and I IFNs, which disrupt sperm formation and transport, ultimately resulting in male infertility. Inducing orchitis with poly(I:C), a dsRNA viral mimic, leads to the secretion of many inflammatory factors, which is consistent with previous studies (Punab et al. 2017). Poly(I:C) activates TLR3 and IPS-1 signaling pathways, resulting in the production of high levels of pro-inflammatory cytokines and I IFNs, which disrupt the integrity of the BTB (Zhu et al. 2013). Additionally, inflammation inhibits testosterone production by downregulating the expression of testosterone synthesis-related proteins Steroidogenic Acute Regulatory Protein (STAR) and MGARP. Inflammation also activates multiple inflammatory signaling pathways, including TNFα and NFκB, in the testes (Fig. 2D). Therefore, it can be concluded that inflammation plays a significant role in male infertility.

Infertility may be related to immuno-metabolic disorders, highlighting the complex relationship between metabolism and the immune system (Martins et al. 2019). Spermatogenesis involves a shift in numerous metabolic processes and the supply of energy, with metabolic factors regulating the function of the endocrine and immune systems in male reproduction (Rato et al. 2012). Studies on mice have indicated that aristolochic acid I lead to testicular toxicity by inhibiting amino acid metabolism, glucose metabolism, fatty acid β-oxidation, and TCA cycling (Cui et al. 2019; Ding et al. 2022). Furthermore, research has demonstrated the significance of cystine, arginine, histidine, and valine in the testes as crucial energy substrates for Sertoli cells, thereby playing a vital role in the energy supply needed for spermatogenesis and the production and maturation of spermatocytes and spermatozoa (Di Fiore et al. 2016). In this study, metabolic pathways were analyzed, falling under 14 different categories, including taurine and hypotaurine metabolism, 2-oxocarboxylic acid metabolism, valine, leucine, and isoleucine biosynthesis, as well as ether lipid and glyceride metabolism (Fig. 3G). Consequently, poly(I:C) primarily affects amino acid and lipid synthesis and metabolism in the mouse testis, leading to disturbances in the levels of endogenous metabolites and subsequently impacting the normal spermatogenesis process.

**Fig. 5:**
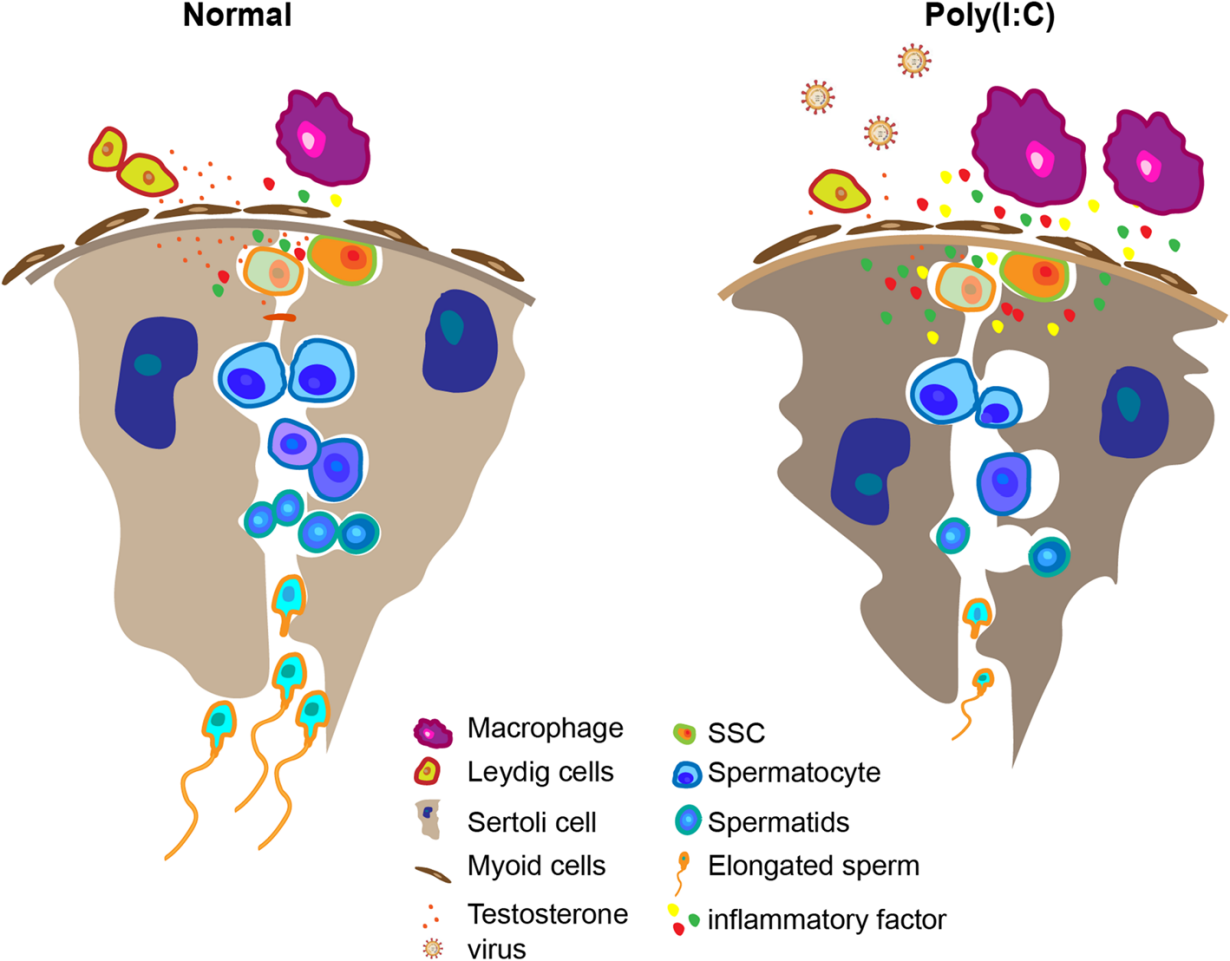
Role of Poly(I:C) in testis. The male reproductive system has a normal immune response regulatory mechanism, but bacteria and viruses can also invade the testis to form orchitis and epididymitis. The study found that Poly(I:C) mainly inhibited the expression of testosterone synthesis, and affected the synthesis and metabolism of amino acids and lipids in the testis. It provides the mechanisms by which RNA virus infection impairs testicular function and offers a theoretical basis for future studies on the immune homeostasis of male reproduction

This study used multi-omics analysis to examine the effects of poly(I:C) infection on protein levels and metabolic levels in the male reproductive system. The analysis revealed the activation of immune pathways and metabolic disorders as the primary contributors to sperm abnormalities. Therefore, this study establishes a foundation for understanding testicular immune homeostasis and offers potential treatments for viral orchitis by targeting inflammatory pathways or supplementing metabolites (Fig. 5). In conclusion, the findings of this study provide valuable insights into the maintenance of testicular immune health and suggest promising therapeutic strategies for viral-induced orchitis.

## Materials and methods

### Animal management and breeding

H﻿ealthy 8-week-old ICR male mice (Chengdu Dossy Experimental Animal Company, China) were used for animal experiments. They were maintained at 25°C with 70% humidity and a 12 h:12 h light/dark cycle. The weight of the mice was 30-35 g. The treatment of mice and experimental manipulations were carried out strictly under the Northwest A&F University’s policies and procedures for the care and use of laboratory animals.

### Establishment of testicular inflammation model

Poly(I:C) was administered via local injection into the testis of mice, following the methodology described in a previous study (Zhu et al. 2013). To ensure anesthesia, mice were administered pentobarbital sodium at a dosage of 50 mg/kg. The testes of mice were injected with 10 uL of poly(I:C) at a concentration of 10 μg/mL; the control was injected with an equal volume of the phosphate buffer saline (PBS) buffer. All the testes from both sides were collected after 6 h for further analysis.

### HE staining and PAS staining

*HE staining:* The paraffin sections underwent deparaffinization using xylene and alcohol with varying concentration gradients, followed by a 2 min wash in distilled water. Hematoxylin and eosin stains were employed to visualize the nucleus and cytoplasm of the cells, respectively. Subsequently, the sections were dehydrated, sealed.

*PAS staining:* Slices are dewaxed and placed in a periodate solution for 10 min after deparaffinization. A 5 min rinse with running water ensued, followed by a 10 min exposure to Schiff’s reagent and another 5 min rinse with running water. The sections were stained with hematoxylin, dehydrated, blocked, and examined using a microscope to capture the images.

### Quantitative real-time PCR (qRT-PCR) analysis

The qRT-PCR procedure was described as the previously described (Wei et al. 2016). The testis total RNA was extracted using Trizol reagent (TakaRa, Dalian, China) as per the manufacturer’s instructions. Subsequently, the total RNA was reverse transcribed into cDNA using the FastKing RT Kit (With gDNase) (TIANGEN BIOTECH, BEIJING, CO., LTD). For the qRT-PCR analysis, the BioEasy Master Mix (SYBR Green, High ROX) from Hangzhou Bioer Technology Co., Ltd. was utilized. Each reaction system consisted of 10 μL of 2 × SYBR Green Mix, 0.5 μL of forward primer, 0.5 μL of reverse primer, 1 μL of cDNA, and 8 μL of ddH_2_O. The qRT-PCR procedure comprised an initial predenaturation step at 95°C for 1 min, followed by 39 cycles of denaturation at 95°C for 15 s, annealing at 60°C for 15 s, and extension at 70°C for 30 s. Primers for the qRT-PCR analysis were produced by Sangon Biotech Biotechnology (Shanghai, China) based on coding sequences from the National Center for Biotechnology Information (NCBI) GenBank. Information on the qRT–PCR primers used in this study is provided in Table 1.

**Table 1:** Primer sequence and gene number were used in this study

Gene name	Forward primer (5’-3’)	Reverse primer (5’-3’)
m-*Uchl1*	GCTCCTCGGGTTTGTGTC	TCTCCTCCTCCAGCCCTA
m-*Tlr3*	GGGATTGGTGAGTCTGAAGT	AGTGAGCAAGGGAGAATGAG
m-*Caspase3*	GTCTGACTGGAAAGCCGAAAC	GACTGGATGAACCACGACCC
m-*Caspase12*	CTGGCTCTCATCATCTGCAACAA	CGGCCAGCAAACTGCATTAAC
m-*Nlrp3*	CTTTATCCACTGCCGAGAG	AGCTCATCAAAGCCATCC
m-*IL-1β*	TGTCCTGATGAGAGCATCC	AAGGTCCACGGGAAAGAC
m-*IL-6*	GTATGAACAACGATGATGCAC	CTCCAGAAGACCAGAGGAAA
m-*Tnfα*	GCAAAGGGAGAGTGGTCA	CTGGCTCTGTGAGGAAGG
m-*actin*	CCTCACTGTCCACCTTCC	GGGTGTAAAACGCAGCTC

### Immunohistochemical (IHC) and immunofluorescence (IF) staining

The IHC and IF staining procedure was according to the previously described (Zhang et al. 2023).

For IHC staining, the paraffin sections for the citrate antigen repair procedure underwent dewaxing and dehydration. After that, they were washed 3 times in PBS to remove impurities. To eliminate endogenous peroxidase activity, the sections were treated with 3% H_2_O_2_ at room temperature (RT) for 10 min. Following this, the sections were sealed with 10% FBS for 30 min to prevent non-specific binding. The primary antibody was then incubated overnight at 4°C. Next, to remove any unbound primary antibody, the sections were washed 3 times in PBS for 2 min each. Subsequently, a horseradish peroxidase-labeled secondary antibody was incubated at RT for 1 hour, followed by three rinses in PBS to remove any excess secondary antibody. To visualize the staining, a 3,3’-Diaminobenzidine (DAB) chromogenic solution was applied. The tissue slices were then counterstained with hematoxylin to visualize the nuclei. Finally, after undergoing gradient alcohol transparency and dehydration, the sections were sealed to preserve the staining. Antibodies and reagents used in the study included: nuclear factor-k-gene binding (NFκB) (Proteintech, 10,745-1-AP, China), interleukin 6 (IL-6) (Proteintech, 21,865-1-AP, China).

For IF, the primary antibody (ZO-1, Proteintech, 21,773-1-AP, China) was diluted from 1:200 in 5% BSA with 0.1% Triton X-100 following the manufacturer’s suggestions, and incubated with sections overnight at 4°C. The sections were then washed with PBS three times. The secondary antibody was diluted 1:400 and incubated for 1 h at RT, followed by washing with PBS. Cell nuclei were stained with DAPI and blocked with coverslips. Images were taken on an EVOS FL fluorescence microscope.

### TUNEL staining

TUNEL staining was performed using a TUNEL staining kit following the manufacturer’s instructions (Beyotime, Shanghai, China). Paraffin sections were deparaffinized by immersing them in xylene, gradient alcohol, and distilled water for 2 min. After deparaffinization, the sections were washed three times with PBS. Subsequently, 20 μg/mL DNase-free proteinase K (20 mg/mL) was added dropwise, and the sections were incubated for 25 min at 25°C. Following the incubation, the sections underwent another round of washing with PBS. Then, the tissue was exposed to 50 μL TUNEL assay solution for 1 h at 37°C, with light being shielded. After this step, the sections were washed three times with PBS. Finally, the slices were sealed using an anti-fluorescence quenching-DAPI 2-in-1 sealer before being examined under a fluorescence microscope.

### Proteomics sequencing and LC-MS/MS analysis

A quantity of 100 mg of testis tissue was introduced into a 1 mL lysis buffer consisting of SDT (4% SDS, 100 mM Tris-HCl, 1 mM dithiothreitol, pH 7.6). The protein content was determined using the BCA Protein Assay Kit (Bio-Rad, USA). 200 μg of protein lysate was obtained from each sample and subjected to trypsinization using the filter-aided sample preparation method (Wiśniewski et al. 2009). The resulting peptide digests from each sample were purified using C18 Cartridges (Empore™ SPE Cartridges C18 (standard density), bed I.D. 7 mm, volume 3 mL, Sigma), concentrated through vacuum centrifugation, and reconstituted in 40 μL of 0.1% (v/v) formic acid. The same procedure was used for the control group.

LC-MS/MS analysis was conducted using a timsTOF Pro mass spectrometer (Bruker) coupled to Nanoelute (Bruker Daltonics) for 60/120/240 minutes. The peptides were loaded onto a reverse phase trap column (Thermo Scientific Acclaim PepMap100, 100 μm*2 cm, nanoViper C18) connected to the C18-reversed phase analytical column (Thermo Scientific Easy Column, 10 cm long, 75 μm inner diameter, 3 μm resin) in buffer A (0.1% Formic acid). Separation was achieved with a linear gradient of buffer B (84% acetonitrile and 0.1% Formic acid) at a flow rate of 300 nl/min controlled by IntelliFlow technology. The mass spectrometer operated in positive ion mode, collecting ion mobility MS spectra in the mass range of m/z 100-1700 and 1/k0 of 0.6 to 1.6. Additionally, 10 cycles of PASEF MS/MS were performed with a target intensity of 1.5 k and a threshold of 2500. Active exclusion was enabled with a release time of 0.4 minutes. The MS raw data for each sample were combined and searched using the MaxQuant 1.5.3.17 software for identification and quantitation analysis. These experimental procedures and data analysis were performed by Applied Protein Technology Co., (Shanghai, China).

### Metabolomics sequencing and LC-MS/MS analysis

Metabolites were isolated from poly(I:C) and control (CON) testicular tissues. The isolation process involved homogenization of the tissues in a 2 mL tube with water and ceramic beads. The extraction of metabolites was carried out through centrifugation using a 1:1 mixture of methanol and acetonitrile. After that, the supernatant was dried and re-dissolved in a 1:1 acetonitrile-water solution before being injected for LC-MS analysis by Applied Protein Technology Co., Shanghai, China. To process the original MS data, Proteo Wizard MSConvert was used to convert the data to MzXML files, which were then imported into the XCMS application. The CAMERA tool was employed to annotate isotopes and adducts. The generated data underwent sum-normalization and were assessed using the ropes R package for further analysis. The selection of potential metabolites was based on the variable importance in the projection (VIP) values and the Student’s t-test, with a threshold of VIP > 1 and *p* value < 0.05 indicating significant metabolites changes. To establish the relationship between two variables, Pearson’s correlation analysis was conducted.

### Western blotting

The testicular tissue suspension is collected and lysed with RIPA buffer on ice for 25 min, then centrifuged at 12,000 rpm for 12 min at 4°C. The supernatant is treated with 5 × SDS loading buffer for 10 min at 100°C, and the proteins are separated by 12% gel electrophoresis. The polyvinylidene fluoride (PVDF) membrane was closed with 8% skim milk, followed by primary and secondary antibody incubation. The position of the bands was observed using a chemiluminescence imaging system (ZY058176, Tanon-4200, China). Antibodies and reagents used in the study included: Aldob (Immunoway, YT0192, China), 1:1000, rabbit, Anxa1 (Immunoway, YT0234, China), 1:1000, rabbit, Irgm (Immunoway, YN2468, China), 1:1000, rabbit, Tssk6 (Immunoway, YT8050, China), 1:1000, rabbit, β-actin (ABclonal Technology, AC038, China), 1:8000, rabbit.

### Enzyme-linked immunosorbent assay (ELISA)

Blood samples were derived from CON and poly(I:C) mice, and serum was obtained by centrifugation at 2000 rpm for 5 min at 4°C. Testosterone (T) levels were measured using an ELISA Assay Kit (FANKEL, China, Testosterone F2569-B) in accordance with the manufacturer’s instructions.

### Statistical analysis

Experimental data was represented using mean ± standard deviation, and the statistical significance of the difference between the data was determined using the Student’s t-test (Excel, Microsoft 2007). The significance levels were denoted as *p* < 0.05 (*), *p* < 0.01 (**), and *p* < 0.001 (***). Data analysis was carried out using GraphPad Prism software.

### Supplementary Information

**Additional file 1: Figure S1.** Metabolomic analysis of Poly(I:C)-induced testicular inflammation. (A). The volcano plot was generated for significantly different metabolites in negative ion mode; (B). The heatmap was created to cluster significant difference metabolites in negative ion mode; (C). The correlation between significant difference metabolites in negative ion mode was visualized using a correlation heatmap; (D). Negative ion mode chordal plot; (E). The network correlation between metabolites in the negative ion mode to depict the correlation between different metabolites.

## Data Availability

The datasets presented in this study can be found in online repositories. The names of the repository/repositories and accession number(s) can be found below: (https://proteomecentral.proteomexchange.org/cgi/GetDataset?ID=PXD045748).

## Abbreviations

*SSCs*: Spermatogonial stem cells
*BTB*: Blood-testicular barrier
*BBB*: Blood-brain barrier
*BPB*: Blood-fetal barrier
*ZIKV*: Zika virus
*HIV*: Human immunodeficiency virus
*MuV*: mumps virus
*NCBI*: National Center for Biotechnology Information
*BP*: Biological Process
*MF*: Molecular Function
*CC*: Cellular Component
*PPI*: Protein-protein interaction network
*DAS*: Differential Abundance Score
*PBS*: Phosphate buffer saline
*RT*: Room temperature
*DAB* 3: 3’-Diaminobenzidine
*NFκB*: Nuclear factor-k-gene binding
*IL-6*: Interleukin 6
*Tlr3*: toll-like receptor 3
*Uchl1*: Ubiquitin C-Terminal Hydrolase L1
*Nlrp3*: NOD-like receptor thermal protein domain associated protein 3
*IL-1β*: Interleukin-1β
*Tnfα*: Tumor necrosis factor alpha
*STAR*: Steroidogenic acute regulatory protein
