# Supplementary material for: A multi-omics analysis of viral nucleic acid poly(I:C) responses to mammalian testicular stimulation
Source: Stress Biol. 2024 Feb 1;4(1):9. doi: 10.1007/s44154-023-00146-6 (PMC10834394; doi:10.1007/s44154-023-00146-6)
Supplement: Supplementary file 1 — Additional file 1: Figure S1. Metabolomic analysis of Poly(I:C)-induced testicular inflammation. (A). The volcano plot was generated for significantly different metabolites in negative ion mode; (B). The heatmap was created to cluster significant difference metabolites in negative ion mode; (C). The correlation between significant difference metabolites in negative ion mode was visualized using a correlation heatmap; (D). Negative ion mode chordal plot; (E). The network correlation between metabolites in the negative ion mode to depict the correlation between different metabolites. [file 44154_2023_146_MOESM1_ESM.docx]

**Title: A multi-omics analysis of viral nucleic acid poly(I:C) responses to mammalian testicular stimulation**

**Author full names**: Donghui Yang^1#^, Wenping Wu^1#^, Qizhong Lu^2^, Yaling Mou^1^, Wenbo Chen^1^, Shicheng Wan^1^, Mengfei Zhang^1^, Congliang Wang^1^, Xiaomin Du^3^, Na Li^1^, Jinlian Hua^1*^

**Institutional addresses:**

^1^College of Veterinary Medicine, Shaanxi Centre of Stem Cells Engineering & Technology, Northwest A&F University, Yangling, Shaanxi, 712100, China.

^2^State Key Laboratory of Biotherapy and Cancer Center, West China Hospital, Sichuan University, Chengdu, 610041, China.

^3^Shaanxi Provincial Engineering and Technology Research Center of Cashmere Goats, College of Life Sciences, Yulin University, Yulin, Shaanxi, 719000, China.

*****Corresponding author information:

*Jinlian Hua

College of Veterinary Medicine, Northwest A&F University, Shaanxi Centre of Stem Cells Engineering & Technology

No. 3rd, Taicheng Road, Yangling, Shaanxi, 712100, China

E-mail: [jinlianhua@nwsuaf.edu.cn](mailto:jinlianhua@nwsuaf.edu.cn)

# Donghui Yang and Wenping Wu contributed equally to this manuscript.


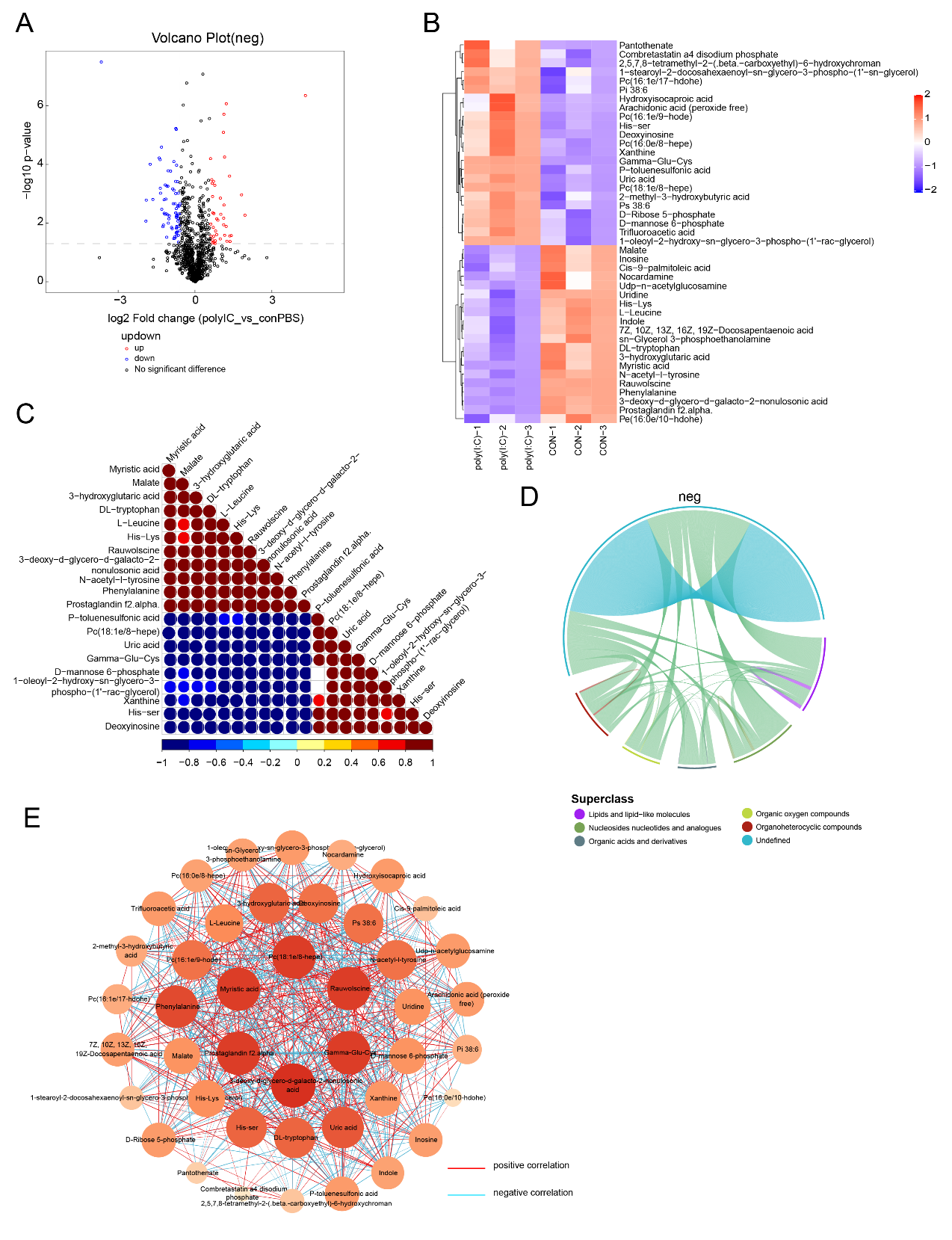


**Fig. S1 Metabolomic analysis of Poly(I:C)-induced testicular inflammation**

(A). The volcano plot was generated for significantly different metabolites in negative ion mode; (B). The heatmap was created to cluster significant difference metabolites in negative ion mode; (C). The correlation between significant difference metabolites in negative ion mode was visualized using a correlation heatmap; (D). Negative ion mode chordal plot; (E). The network correlation between metabolites in the negative ion mode to depict the correlation between different metabolites.
